# Determinants of hand pulse wave velocity and hand pulse transit time in healthy adults

**DOI:** 10.1038/s41598-024-60927-5

**Published:** 2024-05-02

**Authors:** Yung-Sheng Chen, Wan-An Lu, Ling-Yen Hsu, Cheng-Deng Kuo

**Affiliations:** 1 Department of Exercise and Health Sciences, University of Taipei, Taipei, 111 Taiwan; 2 Exercise and Health Promotion Association, New Taipei City, 241 Taiwan; 3 Tanyu Research Laboratory, Taipei, 112 Taiwan; 4 College of LOHAS Industry, Fo-Guang University, Yilan, 262 Taiwan; 5 Institute of Traditional Medicine, National Yang-Ming Chiao-Tung University School of Medicine, Taipei, 112 Taiwan; 6 Department of Internal Medicine, Taipei Veterans General Hospital Hsinchu Branch, Hsinchu County, 310 Taiwan; 7 Leadtek Research Inc., New Taipei City, 235 Taiwan

**Keywords:** Pulse wave velocity, Pulse transit time, Radial artery, Arterial stiffness, Peripheral blood vessel, Cardiology, Engineering

## Abstract

Arterial pulse wave velocity (PWV) is recognized as a convenient method to assess peripheral vascular stiffness. This study explored the clinical characteristics of hand PWV (hPWV) and hand pulse transit time (hPTT) in healthy adults (sixty males = 42.4 ± 13.9 yrs; sixty-four females = 42.8 ± 13.9 yrs) voluntarily participated in this study. The arterial pulse waveform and the anatomical distance from the radial styloid process to the tip of the middle finger of both hands were recorded in the sitting position. The hPWV was calculated as the traversed distance divided by hPTT between those two points. Male subjects showed significantly greater hPWV, systolic blood pressure, and pulse pressure than age-matched female subjects, while the hPTT was not significantly different between genders. Multiple linear regression analysis showed that gender is a common determinant of hPWV and hPTT, and that age and heart rate (HR) were negatively correlated with hPWV and hPTT, respectively. We conclude that male subjects have greater hPWV than female subjects. Ageing is associated with decreased hPWV, while increased HR is associated with a smaller hPTT. The hPWV and hPTT might be used as non-invasive indices to characterise the ageing and arterial stiffness of peripheral blood vessels.

## Introduction

Clinical assessment of arterial stiffness, as measured by pulse wave velocity (PWV), is gaining increasing interest owing to the recognition of PWV as an influential factor in the prognosis of hypertension and an independent predictor of cardiovascular and all-cause mortality. The strength of using PWV is its non-invasiveness and ease of use, which is achieved by measuring two spots in the path of the arterial pulse. In traditional medicine, the practitioners often diagnose the patients' health and disease by palpating their wrist pulses^1^. The PWV has been used extensively to assess vascular functions in clinical settings^2^.

The PWV is a physiological index of arterial stiffness in the vascular system^3^. Arterial stiffness is influenced by structural and functional changes in the arteries, such as the composition of the arterial wall, left ventricular afterload, vascular resistance, etc^4^. Other factors, such as age^5,6^, gender^7,8^, and anthropometric characteristics^9,10^, are also associated with PWV. It has been demonstrated that increased PWV is associated with cardiovascular risks, including hypercholesterolemia^11^, diabetes mellitus^12,13^, and sedentary lifestyle^14^. The intensity and speed of pulse waves in the wrist radial artery are strongly associated with blood pressure (BP), and heart rate (HR) in patients with hypertension^15^. The time that the pulse requires to propagate between two spots is the pulse transit time (PTT) for the distance between those two spots. The underlying mechanisms determining PTT are related to cardiac contractility, arterial compliance, total peripheral resistance, and blood constituents^16^.

Several valid and reliable methods for the measurement of PWV have been developed, including brachial-ankle PWV (ba-PWV)^7^, carotid-femoral PWV (cf-PWV)^17^, finger-toe PWV (ft-PWV)^18^, carotid-radial PWV (cr-PWV)^19–21^, radial-digital PWV (rd-PWV)^22,23^, and radial-finger PWV (rf-PWV)^24^. The cf-PWV has been recognized as the primary option and gold standard for clinical evaluation of vascular health^17^. The ft-PWV correlates significantly with cf-PWV, and is a promising means of assessing arterial stiffness^18^. The physiological and structural discrepancies at different locations of the arterial tree limit the direct comparison among other methods (e.g., artery size, diameter of the artery, and peripheral resistance).

The rd-PWV^22,23^ or rf-PWV^24^ has been developed to evaluate arterial stiffness of peripheral small conduit arteries. The rd-PWV or rf-PWV is calculated using the differences between radial-digital distance and radial-finger distance, and respective PTT using the foot-to-foot method to assess the regional arterial stiffness of small conduit arteries of the hand in healthy adults. Using rf-PWV, it was found that the stiffness of small conduit arteries increases after nitroglycerin administration^24^.

In this study, we introduced a simple way of calculating the hand PWV (hPWV) and hand PTT (hPTT) measured between the radial wrist artery and the fingertip of the middle finger in healthy adults. Additionally, the anthropometric variables including age, body height, body weight, and BMI, and hemodynamic variables including systolic blood pressure (SBP), diastolic blood pressure (DBP), pulse pressure (PP), mean arterial blood pressure (MABP), and resting heart rate (HR) were examined to evaluate the association of these factors with the hPWV and hPTT in healthy adults.

## Results

### Physical and hemodynamic characteristics

**Figure 1:**
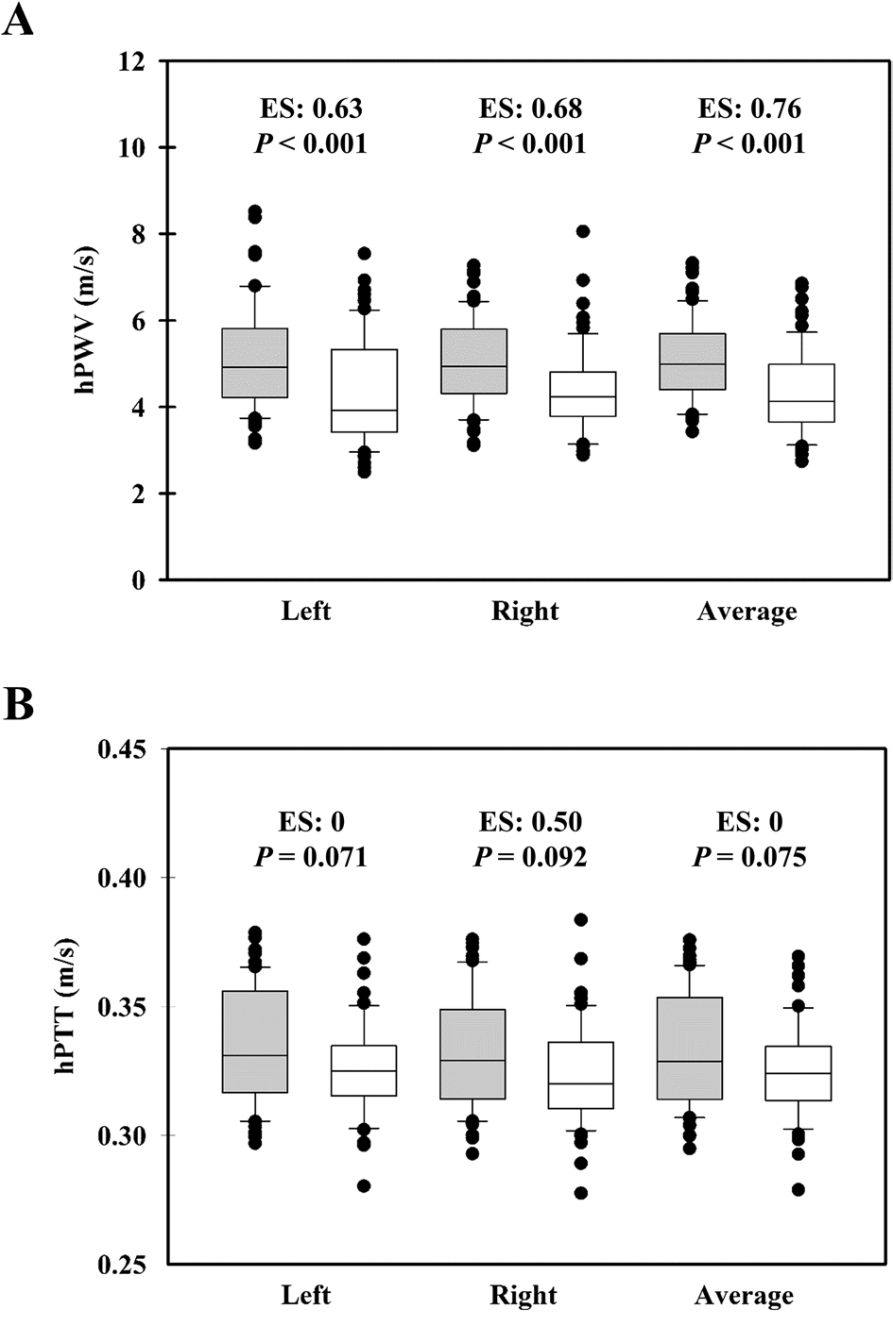
Comparison of hand pulse wave velocity (hPWV) (**A**) and hand pulse transit time (hPTT) (**B**) between male and female subjects. The gray box plot indicates data recorded from male adults, while the white box plot indicates data recorded from female adults. *hPWV* hand pulse wave velocity, *hPTT* hand pulse transit time, *ES* effect size, *s* second, *m/s* meter per second.

Table 1 shows the physical and hemodynamic profile of the participants. The distance from the styloid process of the radius to the fingertip of the middle finger is significantly longer in male subjects’ left hand (p < 0.001, effect size (ES) = 0.71), right hand (*p* < 0.001, ES = 0.80), average hands (*p* < 0.001, ES = 0.86), compared to female subjects. The body height (*p* < 0.001, ES = 1.82), body weight (*p* < 0.001, ES = 1.48), BMI (*p* < 0.001, ES = 0.68), SBP (*p* < 0.001, ES = 0.49), and PP (*p* < 0.001, ES = 0.68) of male subjects were greater than those of female subjects. Figure 1 shows that male subjects (gray boxes) had greater hPWV than age-matched female subjects (white boxes)(left hand: *p* < 0.001, ES = 0.63; right hand: *p* < 0.001, ES = 0.68; and average hands: *p* < 0.001, ES = 0.76). However, the hPTT was not significantly different between genders.

**Table 1:** Physical and hemodynamic characteristics of study participants.

Parameters	Male (n = 60)	Female (n = 64)	Effect size	p-value
Age (yrs)	42.4 ± 13.9	42.8 ± 13.9	− 0.03 (− 0.38;0.33), Trivial	0.889
Body height (m)	1.69 ± 0.06	1.58 ± 0.06	1.82 (1.41;2.25), Large	< 0.001
Body weight (kg)	67.5 ± 10.6	53.8 ± 7.8	1.48 (1.09;1.89), Large	< 0.001
BMI (kg/m^2^)	23.59 ± 3.03	21.52 ± 3.04	0.68 (0.32;1.04), Moderate	< 0.001
Left hand *Δx* (cm)	15.83 (14.37;19.01)	12.98 (11.18;17.12)	0.71 (0.35;1.08), Moderate	< 0.001
Right hand *Δx* (cm)	16.42 (13.96;18.92)	13.75 (12.06;15.33)	0.80 (0.44;1.17), Moderate	< 0.001
Average *Δx* (cm)	16.50 (14.34;19.03)	13.03 (14.34;15.94)	0.86 (0.50;1.23), Moderate	< 0.001
SBP (mmHg)	119 (110;128)	110 (104;123)	0.49 (0.13;0.85), Small	0.004
DBP (mmHg)	74 (68;82)	73 (67;80)	0.11 (− 0.24; 0.47), Trivial	0.485
PP (mmHg)	44 (37;49)	38 (35;43)	0.68 (0.32; 1.04), Moderate	< 0.001
MABP (mmHg)	88 (83;98)	85 (79;95)	0.27 (− 0.09;0.62), Small	0.116
HR (bpm)	73 (64;80)	75 (66;78)	− 0.09 (− 0.44;0.26), Trivial	0.635

Data are presented as mean and standard deviation (SD) or median and interquartile range (IQR, 25–75%).

*BMI* body mass index, *Δx* the distance from the radial styloid process to the fingertip of the middle finger, *SBP* systolic blood pressure, *DBP* diastolic blood pressure, *PP* pulse pressure, *MABP* mean arterial blood pressure, *HR* heart rate, *yrs* years, *m* meter, *kg* kilogram, *m*^*2*^ meter square, *mmHg* millimeter of mercury, *bpm* beat per minute.

### Determinants of *hPWV* and *hPTT*

If the gender value is assigned 1 for male subjects and 0 for female subjects, then multiple linear regression analysis showed that the hPWV of either hand and the average hPWV of both hands have a positive correlation with gender and a negative correlation with age. The hPTT of either hand and the average hPTT of both hands have a positive correlation with gender and a negative correlation with HR. Other physical and hemodynamic variables such as body height, body weight, BMI, SBP, DBP, and MABP are not correlated significantly with either hPWV or hPTT of either hand. The linear equations of hPWV and hPTT of both hands are (n = 124):

Left hPWV = 5.914 + 0.755 * Gender - 0.0371 * Age (*r*^2^ = 0. 254, *p* < 0.001)

Right hPWV = 5.269 + 0.678 * Gender - 0.0215 * Age (*r*^2^ = 0.187, *p* < 0.001)

Average hPWV = 5.592 + 0.717 * Gender - 0.0293 * Age (*r*^2^ = 282, *p* < 0.001)

Left hPTT = 0.386 + 0.00675 * Gender - 0.000815 * HR (*r*^2^ = 0.166, *p* < 0.001)

Right hPTT = 0.373 + 0.00737 * Gender - 0.000676 * HR (*r*^2^ = 0.127, *p* < 0.001)

Average hPTT = 0.380 + 0.00706 * Gender - 0.000746 * HR (*r*^2^ = 0.153, *p* < 0.001)

It is evident that gender is the common determinant of hPWV and hPTT; age is another determinant of hPWV, while HR is another determinant of hPTT. The positive correlations between hPWV and gender, and between hPTT and gender, indicate that male subject have larger hPWV and hPTT. The negative correlation between hPWV and age indicates that older age is associated with smaller hPWV. Similarly, the negative correlation between hPTT and HR indicates that a faster HR is associated with a smaller hPTT.

## Discussion

The main finding in the present study is that there are significant differences in SBP, PP, hPWV, and hPTT between male subjects and female subjects. In this study, we performed statistical analysis with both genders incorporated in the same regression analysis so that the common features of male subjects and female subjects could be displayed, and the two linear regression equations for males and females could be combined into one. We found that male subjects have greater hPWV than female subjects. Age and HR are negatively correlated with hPWV and hPTT.

The hPWV of male subjects is greater than age-matched female subjects in this study, in consistence with the well-known finding that PWV is different between male and female adults^26^. Tomiyama et al.^7^ showed that aortic-brachial PWV is higher in males than in age-matched females. London et al.^27^ reported lower carotid-radial PWV and femoral-tibial PWV in premenopausal women than in age-matched men. The underlying mechanisms involved in the difference in PWV between male subjects and female subjects are complex, but all related to hormonal regulation, sympathetic tone on vascular functions, and cardiac activities. It has been shown that sex hormones have modulatory effects on vascular ageing^28^. The PWV can be used as a biomarker to indicate normal or abnormal status of peripheral blood vessels, and be used in cardiovascular disease risk stratification, assessment of hypertension and vascular stiffness, and assessment of therapeutic effects in clinical studies^29^.

To investigate the effect of ageing on hPWV, we recruited participants aged between 21 and 66 years. Multiple linear regression analysis revealed that age is a predominated factor that influences the hPWV. Our finding aligns with recent studies showing that the carotid-radial^20^, brachial-radial^9^, and finger-toe PWV^18,30^ are negatively correlated to age. The increase in the thickness of the arterial wall and loss of elastin and its replacement with collagen may contribute to the smaller hPWV in elderly adults. Age-related decreases in cardiac function might be another factor contributing to the smaller hPWV in aged people.

It has been reported that PTT is strongly associated with HR^31^. Increased HR is related to increased aortic stiffness in participants with stiffer aortas, regardless of BP and other risk factors and participants' characteristics^32^. In this study, we found that hPTT is negatively correlated with HR. A faster HR is associated with a small hPTT. This is conceivable because HR is inversely associated with PTT.

The difference between the methodologies utilized by Obeid et al.^22^ and the present study lies in the indirect/direct measurement of PWV and PTT. In the study of Obeid et al.^22^, the PTT from the carotid to the radial location and from the carotid to the digital location are measured indirectly; and the differences between carotid-digital distance and carotid-radial distance and the respective transit times are used as the PTT to calculate the PWV. In contrast, in the present study the hPTT and the distance from the radial styloid process to the fingertip of the middle finger are directly measured to calculate the hPWV. Since the actual traversed distance of pulse wave from the heart to the distal part of fingers/toes is not feasible to measure in vivo, the direct measurement of hPTT and hPWV in the present study might provide a friendly, convenient, and easy-to-use approach for daily evaluation of arterial health.

In the calculation of rd-PWV in the study of Obeid et al., the piezoelectric sensors are placed both at the radial artery and the index (left)^22^. The transit time between two simulated waveforms was calculated using the foot-to-foot method using MATLAB-based maximum second-derivative algorithm and the intersecting-tangent algorithms to identify the foot of the pulse wave. Though these two methods give similar results of PTT, the results of the foot-to-foot method might be influenced by noisy elements in the signals. Therefore, a low-pass filter of 49 Hz must be applied to all waveforms to block all frequencies below 49Hz^22^. To overcome this limitation of the foot-to-foot method, we adopt the peak-to-peak method used in measuring beat-to-beat intervals in many studies of heart rate variability^33^ to measure the PTT. The time interval between the peak of the pulse waves at the radial styloid process and that at the fingertip of the middle finger is the hPTT in this study. An apparent advantage of the peak-to-peak method is that the measured PTT will not be affected by the noisy elements in the pulse waveforms.

In a previous study, the arterial pulse wave (APW) characteristics of the right hand, left hand, and left foot were recorded for power spectral analysis^19^. The present study directly measured the hPTT and the distance from the radial styloid process to the fingertip of the middle finger to calculate the hPWV. Since the APW has been used to assess cardiovascular functions, the direct measurement of hPTT and hPWV in the present study might provide another non-invasive method to evaluate peripheral arterial functions.

The main limitation of the present study was that indirect measurement of PWV was not performed in this study. A comparison of the PWV values between direct and indirect measurements is required in future studies. The second limitation of the present study was that only healthy adults were recruited in this study. To fully understand the clinical meaning and usefulness of hPWV and hPTT, it is necessary to compare the hPWV and hPTT of healthy adults with those patients with cardiovascular illness or other major illnesses. Further studies are needed to elucidate the prognostic implications of hPWV and hPTT in various cardiovascular diseases or other major illnesses.

Male subjects have greater hPWV than age-matched females, while the hPTT is not significantly different between genders. Gender is the common determinant of hPWV and hPTT. Aging is associated with decreased hPWV, whereas increased HR is associated with a smaller hPTT. Both hPWV and hPTT might be useful in characterizing ageing and arterial stiffness of peripheral blood vessels.

## Methods

### Participants

Sixty males (age = 42.4 ± 13.9 years; body height = 1.79 ± 0.06 m; body weight = 67.5 ± 10.6 kg) and sixty-four females (age = 42.8 ± 13.9; body height = 1.58 ± 0.06 m; body weight = 53.8 ± 7.8 kg) voluntarily participated in this study (Table 1). Inclusion criteria included: (1) healthy lifestyle; (2) age greater than 20 years old. Exclusion criteria included: (1) undertaking clinical treatments for cardiovascular diseases; (2) hypertension (systolic blood pressure, SBP > 140 mmHg and diastolic blood pressure, DBP > 90 mmHg; (3) body mass index (BMI) > 30 kg/m^2^; (4) presence of atrial fibrillation, extremity tremor, deformation of limbs or digits, and history of cerebral vascular accident. This study was approved by the Institutional Review Board of the Taipei Veterans General Hospital (TVGHIRB-98-01-28A). All participants gave their written informed consent form prior to participation. The experimental protocol for human studies was performed in accordance with the Declaration of Helsinki.

### Experimental procedure

**Figure 2:**
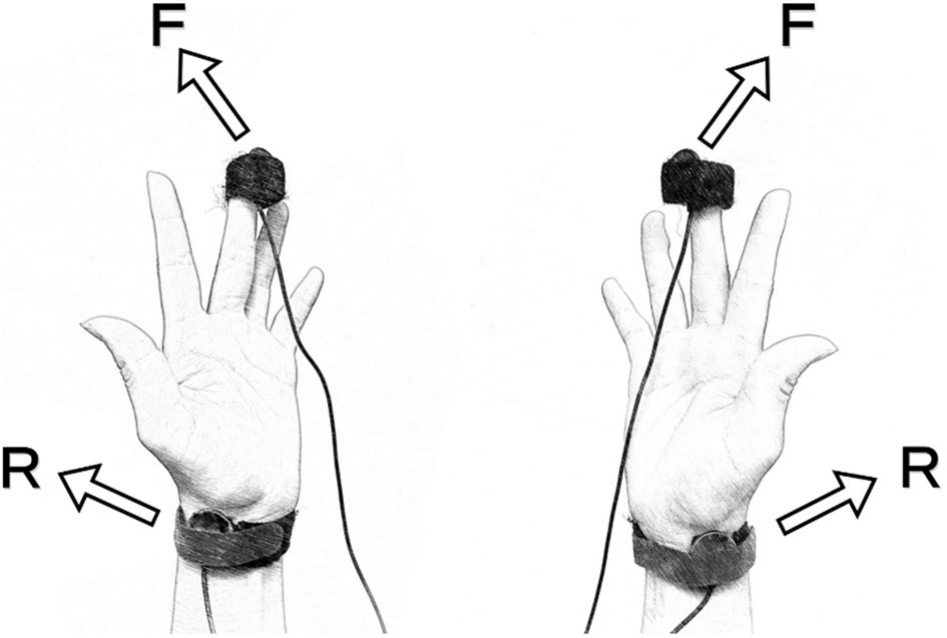
Schematic illustration of the experimental set-up. The distance from the styloid process of the radius to the middle fingertip was denoted as *Δx*. The time interval between the peak of the pulse waves detected at the radial styloid process of the radius (denoted as R) and the middle fingertip (denoted as F) is the hPTT. The hPWV was calculated using the formula: hPWV = *Δx*/hPTT.

A cross-sectional comparison was used in this study. All participants completed the study in the afternoon to prevent circadian rhythm on vascular functions. The participants were asked to prevent caffeinated intake 24 h prior to the experiment visit. During the study, the participants were requested to rest in the sitting position on a comfortable couch for 5 min, then the BP was measured using an automated BP monitor (Omron R3, Omron Healthcare Co., Tokyo, Japan). The pulse transducers (MLT1010 Piezo-Electric pulse transducer, ADInstruments, Sydney, Australia) were placed at the radial styloid process and the palm side of the fingertip of the middle finger of both hands (Fig. 2). The hands were positioned with the palms flat, facing up, with arms placed on the table. The arterial pulse waves at the radial styloid process and the tip of the middle fingertip were recorded at the same time using a data acquisition system (ML795 PowerLab/16sp, ADInstruments, Sydney, Australia) with a sampling frequency of 4,000 Hz.

### Data analysis

**Figure 3:**
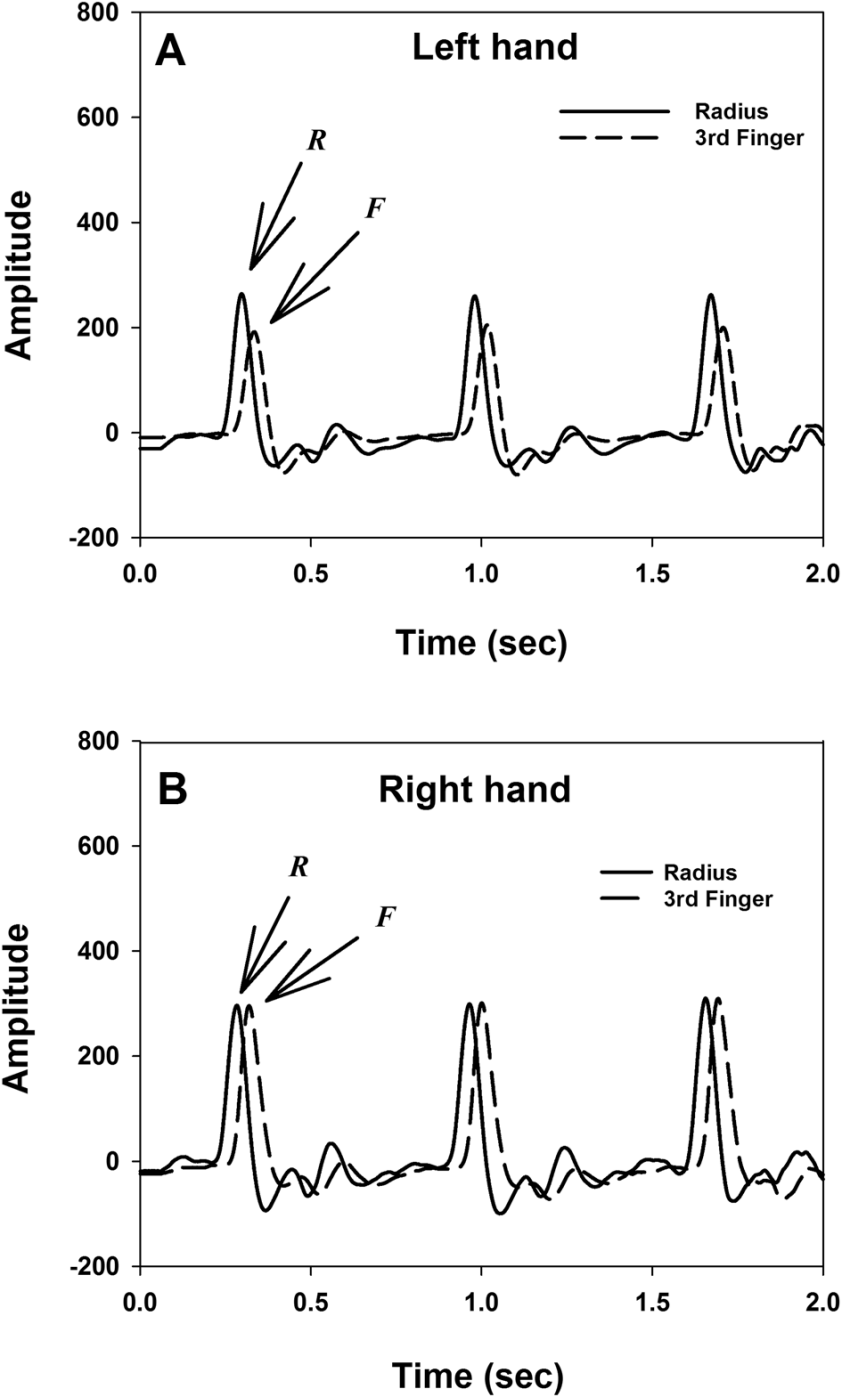
The arterial pulse waves tracings on the left hand (**A**) and right hand (**B**) of a study subject. The arterial pulse wave tracing recorded at the radial styloid process of the radius (R) is presented as a solid line, while the arterial pulse wave tracing recorded at the fingertip of the middle finger (F) is presented as a dash line. *hPWV* hand pulse wave velocity, *hPTT* hand pulse transit time, *s* second.

The time interval between the peaks of the pulse waves detected at the radial styloid process (denoted as R) and at the fingertip of the middle finger (denoted as F) was measured as the hPTT. The distance between those two locations was measured using a commercial measuring tape. The hPWV was calculated using the following formula: hPWV = Δ*x*/hPTT, where Δ*x* is the distance from the radial styloid process to the fingertip of the middle finger. An average value of hPTT and hPWV of 20 consecutive pulses was used as the hPTT and hPWV of the subject. Figure 3 shows the arterial pulse wave tracings recorded at the radial styloid process (R) and the fingertip of middle finger (F) of left and right hands of a representative subject to depict how is pulse transit time (PTT) measured in this study.

### Statistical analyses

Statistical analysis was performed using SigmaPlot version 13 for Windows (Systat Software, California, USA). When appropriate, descriptive data are presented as mean and standard deviation (SD) or median and interquartile range (IQR, 25%-75%). The normality of the variables was examined using the Kolmogorov–Smirnov test. A paired sample t-test and Mann–Whitney rank sum test were employed to compare parametric and non-parametric variables between male and female adults, respectively. The Cohen *d* standardized difference was used to examine the effect size (ES) of pairwise comparisons. The magnitude level of ES was interpreted as trivial (0.0–0.2), small (0.2–0.6), moderate (0.6–1.2), large (1.2–2.0), and very large (> 2.0)^25^. Multiple linear regression analysis was used to examine the determinants of hPWV and hPTT among anthropometric variables, including age, body height, body weight, and BMI, and hemodynamic variables, including SBP, DBP, PP, MABP, and resting HR. Correlation analysis was used to determine whether the effects under investigation were independent of each other or not. A *p* < 0.05 was set as the limit of significant difference.

## Data Availability

The datasets used and analyzed during the current study are available from the corresponding author upon reasonable request.
